# Debutant iOS app and gene-disease complexities in clinical genomics and precision medicine

**DOI:** 10.1186/s40169-019-0243-8

**Published:** 2019-10-04

**Authors:** Zeeshan Ahmed, Saman Zeeshan, Ruoyun Xiong, Bruce T. Liang

**Affiliations:** 1 Department of Genetics and Genome Sciences, School of Medicine, University of Connecticut Health Center (UConn Health), 263 Farmington Ave, Farmington, CT 06032 USA; 2 Institute for Systems Genomics, University of Connecticut, 263 Farmington Ave, Farmington, CT 06032 USA; 3 The Jackson Laboratory for Genomic Medicine, 10 Discovery Drive, Farmington, CT 06032 USA; 4 Pat and Jim Calhoun Cardiology Center, School of Medicine, UConn Health, 263 Farmington Ave, Farmington, CT 06032 USA

## Abstract

**Background:**

The last decade has seen a dramatic increase in the availability of scientific data, where human-related biological databases have grown not only in count but also in volume, posing unprecedented challenges in data storage, processing, analysis, exchange, and curation. Next generation sequencing (NGS) advancements have facilitated and accelerated the process of identifying genetic variations. Adopting NGS with Whole-Genome and RNA sequencing in a diagnostic context has the potential to improve disease-risk detection in support of precision medicine and drug discovery. Several bioinformatics pipelines have been developed to strengthen variant interpretation by efficiently processing and analyzing sequence data, whereas many published results show how genomics data can be proactively incorporated into medical practices and improve utilization of clinical information. To utilize the wealth of genomics and health, there is a crucial need to generate appropriate gene-disease annotation repositories accessed through modern technology.

**Results:**

Our focus here is to create a comprehensive database with mobile access to actionable genes and classified diseases, considered the foundation for clinical genomics and precision medicine. We present a publicly available iOS app, PAS-Gen, which invites global users to freely download it on iPhone and iPad devices, quickly adopt its easy to use interface, and search for genes and related diseases. PAS-Gen was developed using Swift, XCODE, and PHP scripting that uses Web and MySQL database servers, which includes over 59,000 protein-coding and non-coding genes, and over 90,000 classified gene-disease associations. PAS-Gen is founded on the clinical and scientific premise that easier healthcare and genomics data sharing will accelerate future medical discoveries.

**Conclusions:**

We present a cutting-edge gene-disease database with a smart phone application, integrating information on classified diseases and related genes. The PAS-Gen app will assist researchers, medical practitioners, and pharmacists by providing a broad and view of genes that may be implicated in the likelihood of developing certain diseases. This tool with accelerate users’ abilities to understand the genetic basis of human complex diseases and by assimilating genomic and phenotypic data will support future work to identify gene-specific designer drugs, target precise molecular fingerprints for tumors, suggest appropriate drug therapies, predict individual susceptibility to disease, and diagnose and treat rare illnesses.

## Background

From the beginning of scientific discoveries, it has been central to understand the causes of disease, pain, and senescence. Over the centuries, quests for the answers have led us to take giant leaps. It was only in the last century that the discovery of antibiotics freed us from many of the dreaded diseases of the past. Today, we stand on the threshold of a new medical revolution, just as big and far-reaching. Despite all our scientific knowledge, medicine still faces several critical and conflicting challenges. One of the challenges is the transition from a disease-based model to a patient-oriented approach as much of medicine is still based on symptomatic treatments. Disease classification is routinely derived from different streams of healthcare unit data, which includes imaging, pathology, genomics, electrophysiology, and others [1]. Incorporating genetic information assists in producing individual treatment solutions, rather than what works for the average person, and understanding who is at risk for critical diseases like diabetes, high blood pressure, or cancer. This allows for rapid disease at an early stage, accurate characterization of disease, and preventive measures needed before the disease even appears. Also, timely discovery and association of genetic variants with diseases can help develop a more effective therapy tailored to an individual’s precise genetic makeup and reduces adverse drug reactions. Occasionally, technological advancements in genomics have revolutionized the field with gene number proposition, genetic mapping, data banks, gene-disease maps, catalogues of human genes and genetic disorders, big data, and next generation sequencing (NGS) [2]. As biological data accumulates at larger scales and at exponential rates, with higher-throughput and lower-cost DNA sequencing technologies, it has become essential to develop innovative, smart, and modern bioinformatics applications to help improve research quality. New tools provide a progressive understanding of heterogeneous genomics and clinical findings and facilitate increased clinical utilization of information in these databases and translation to healthcare.

The word “Gene” was introduced over 100 years ago [3], and its meaning has progressively evolved in several scientific directions [4–6]. A gene is a segment of DNA sequence that carries genetic information defining a biological function and can be transferred from parent to offspring [7, 8]. Most human genes have a discontinuous structure, with the protein coding regions, or exons, interrupted by non-coding regions, or introns [9, 10]. For some time, many researchers used a broad estimate of gene count at more than 50,000 genes including 21,000 protein-coding genes [11]. However, this number has repeatedly been overturned with advancements in genetics and genomics research. A major goal of medical genetics is to identify genes that when altered lead to human disease, but not all recognizable DNA sequence alterations result in disease [12]. Most alterations, or mutations, are simple differences called single nucleotide polymorphisms (SNPs) that may not change the expression or coding of a gene, but some specific mutations can change gene instructions, and ultimately create a protein malfunction, which may cause disease. If we can identify which genetic variations are associated with specific diseases, we will be better equipped to find new treatments and even cures.

Today, scientists have identified genetic mutations responsible for thousands of conditions, such as cancer, hypertension, and heart disease that affect millions of people. These associations were not easily deciphered, because they are often impacted by interactions between dozens of different genes, many of which are caused by single gene elements or the environment. To identify the genetic signatures of these complex common elements, scientists may have to profile the genetic signatures of thousands of people, even multiple populations, and not just a few individuals. However, studying the genome and epigenome (chemically-modified genome) [13] has led to the fundamentals of development and progression of human diseases [14], which are characterized as multifactorial, mitochondrial [15], chromosomal [16], and monogenic [17] diseases. All human diseases are maintained by the World Health Organization (WHO) with the standard creation of International Classification of Diseases (ICD) codes. With the emergence of next-generation gene sequencing, numerous databases have surfaced for gene annotation, which claim to provide information about genes and link them to related diseases (e.g., Disease Ontology [18], DiseaseEnhancer [19], DISEASES [20], DisGeNET [21], eDGAR [22], GeneCard [23], GTR [24], MalaCard [25], OMIM [26], miR2Disease [27], HGMD [28], DNetDB [29], ClinVar [30], Orphanet, Gene2Function, etc.), and are accessed through web and desktop interfaces. These databases are useful, but none of them contain up-to-date genome and disease data in a standardized format and accessible through a single application platform.

One platform that has proven to be an efficient tool in several areas including healthcare, is the smartphone application. As smart devices have become increasingly popular, there is still no iOS app publicly available that can provide unified access to genomic databases with easy navigation and free portable access to genes and related diseases for efficient and robust classifications. The reasons could be extensive heterogeneity of clinical and genomic data collection and management, and addressing complexities of implementing an Apple mobile app. Developing such a mobile repository, can assist healthcare providers, researchers, and pharmaceutical companies to integrate their health information systems inter-organizationally, develop clinical decision-support systems for disease state management, perform effective comparisons between studies, and enable the quick identification of patients for inclusion to intervention and observational studies. The objectives of our research is to create a centralized gene-disease database, which not only stores, organizes, and shares data in a structured and searchable manner but also facilitates data retrieval with a smartphone application.

## Implementation

Developing an iOS app is an unorthodox bioinformatics application development process, especially when it is expected to be installed in all models of the available iPhone and iPad devices working with timely and latest versions of operating systems installed. It is even more complex when it needs to connect to the external web-based database servers for data acquisitions utilizing internet resources, with imposed stringent security conditions by the host organization. One of the most difficult and complex tasks of implementing an iOS app connecting a mobile interface via web programmed modules to the database server for data exchange is the integration of all modules developed using different programming languages and processed through different compilers/interpreters on a single platform. This often leads to complicated logical errors that are hard to resolve.

**Fig. 1:**
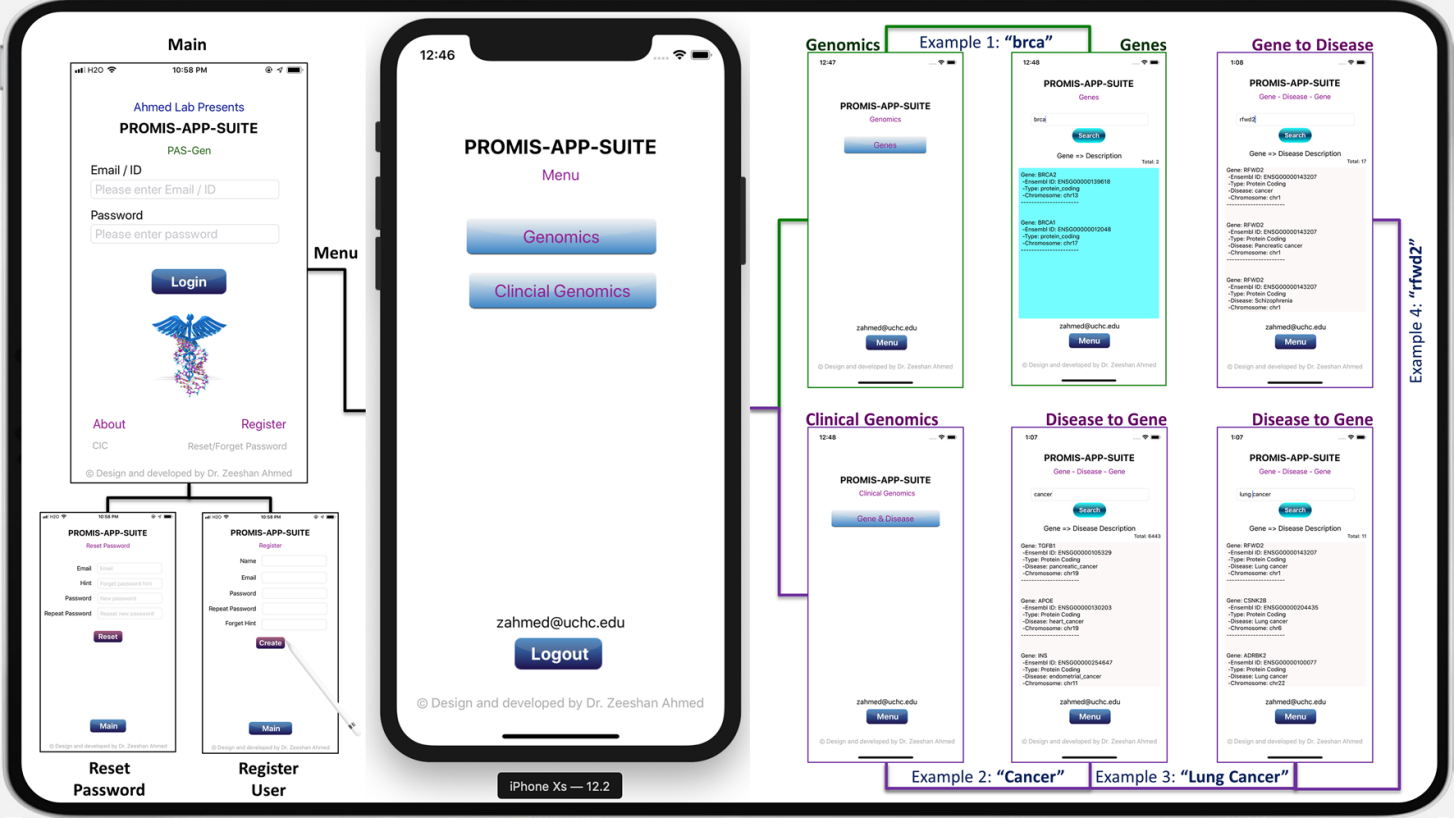
PAS-Gen navigating graphical user interfaces with examples of searched Gene, Gene to Disease, and Disease to Gene results. PAS-Gen (iPhone XS and 8) screen display includes About, Register User, Reset Password, Main, Menu, Genomics, Clinical Genomics, Genes, and Genes and Disease interfaces. Example 1 shows a search by entering an incomplete gene name “BRCA” (BReast CAncer gene) that reveals the for protein coding genes “BRCA1” and “BRCA2” and related details. Example 2 is a search using keyword “cancer” that presents 6443 genes known to be involved in different kinds of cancers. In example 3, a search for a specific disease “lung cancer” resulted in a total of 11 genes and related diseases. Example 4 demonstrates a search for the gene “RFWD2”, and results revealed 17 disease matches including a protein coding gene with Ensembl ID “ENSG00000143207” at Chromosome 1 associated with the disease “Autism”. Detailed results are attached in Additional file 1

**Fig. 2:**
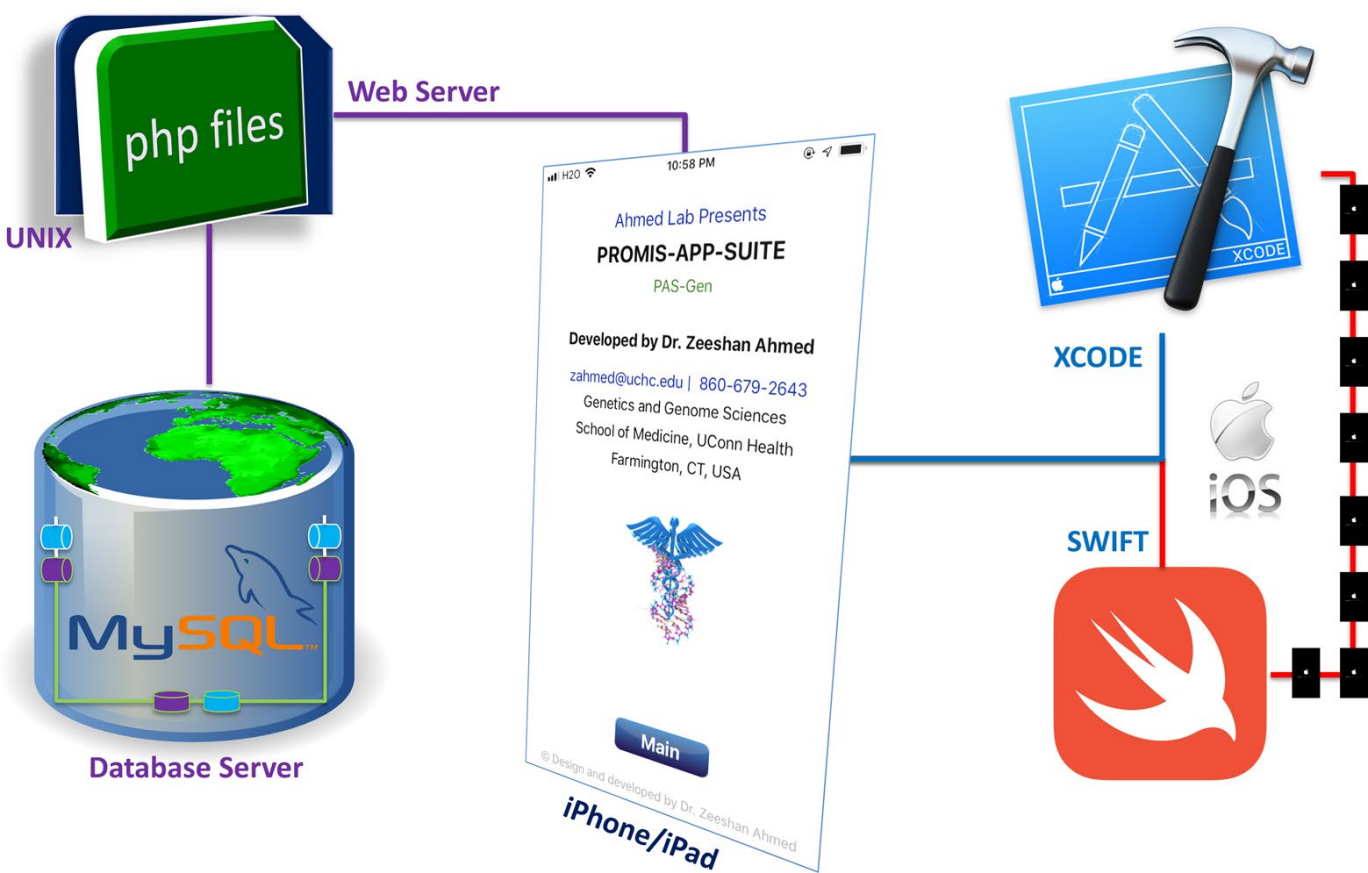
PAS-Gen components design, development, and data flow. PAS-Gen is an iOS app developed with Swift programming language, XCODE integrated development environment for MacOS, MySQL database management system, PHP scripting language, and UNIX-based web and database servers

PROMIS-APP-SUITE (PAS)—Gen (Fig. 1) is an iOS app developed with Swift programming language, using the XCODE (Version 10.2.1 (10E1001)) integrated development environment for MacOS. We designed the human interface of PAS-Gen following Apple’s recommended design principles, which include Aesthetic Integrity, Consistency, Direct Manipulation, Feedback, Metaphors, and User Control. The front end of all the graphical user interfaces (scenes) were designed and connected using XCODE’s built-in Storyboard. The backend of all the screens were programmed in Swift programming language, mainly importing UIKit. The database of PAS-Gen was modelled and implemented within the MySQL database management system, which was publicly hosted via Apache HTTP Server. PAS-Gen database includes human reference genomes collected from different genomics databases worldwide, including ClinVar [30], GeneCards [23], DISEASES [20], HGMD [28], OMIM [26], GTR [24], CNVD [31], Ensembl [32], GenCode [33], Novoseek, Swiss-Prot, LncRNADisease, and Orphanet. None of these databases provide a mobile interface for usage. PAS-Gen design is very flexible, and can accommodate new releases and updates of genes and diseases without requiring its users to install a new version (Fig. 1). Dynamic web-based modules (pages) were developed using the PHP scripting language to facilitate data migration between the iOS app screens and MySQL database server (Fig. 2). The design is based on product line architecture (PLA) [34–36], modelled on the Butterfly model [37, 38], with all major modules implemented following software engineering principles, which are capable of performing individual key roles and can assimilate in a large-scale project. During development, the performance of PAS-Gen was tested using built-in virtual iPhone and iPad kits, and real time iPhone (8 and XS with pre-installed iOS 12.4) and 3^rd^ generation iPad devices. The released, currently available version of PAS-Gen was tested and approved by Apple for meeting expected international standards, which include architecture, user interaction, system capabilities, visual design, icon and images, windows and views, extensions etc.

PAS-Gen graphical interface provides user profile, login, and password management modules, requiring new users to first register by creating an account and login with valid credentials. The major reason for requesting users to create a profile, is to apply security features to the app to track usage and backtrack in case of any trouble, such as a breach or violation. In the future, we plan to implement artificial intelligence and machine learning-based features to help users search data of their interest based on their search history, and having their profile will be extremely useful in such cases. Moreover, a user email address is required to inform on major updates to the app and database. At successful login, users will be directed to the main menu leading to the “Genomics” and “Clinical Genomics” interfaces, with two similarly designed interfaces: “Genes” and “Gene & Disease”. The “Genomics” button leads to the “Genes” interface, which allows users to search for only genes and related information, which includes Gene Name, Ensembl ID, Type, and Chromosome. The “Clinical Genomics” button leads to the “Gene & Disease” interface, which lets users search for related diseases by complete or partial word matching. One important thing to remember while searching for any disease leading to genes is, if the name of the disease consists of multiple words then using underscore “_” instead of space or hyphen is required (e.g., type “Down_Syndrome” for “Down Syndrome” or “Tay_Sachs” for “Tay-Sachs”). PAS-Gen is for non-commercial research and educational use only. It is freely and only available on the App Store for iOS devices, tested and recommended for the iPhone 6, 8, X (XS, MAX), and iPad (2nd and 3rd Generation) mobile devices with iOS version 12.1 or above (Fig. 1).

Further download and project-related details are available at the following web site: https://itunes.apple.com/us/app/pas-gen/id1447766164?ls=1&mt=8.

## Results

**Fig. 3:**
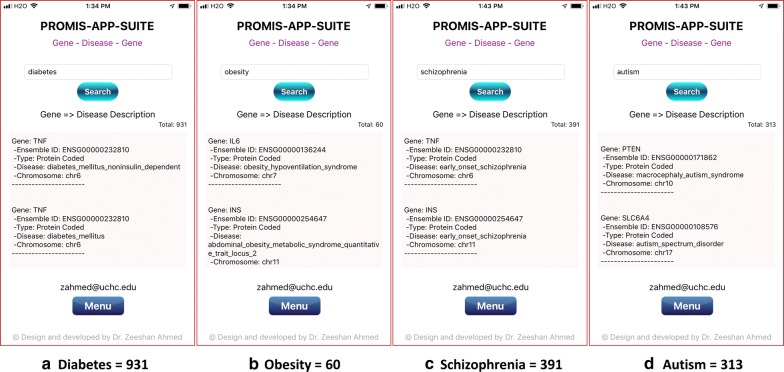
PAS-Gen (iPhone 8) screenshot examples of gene results (top two shown) from searches for the four most common diseases: **a** 931 results for Diabetes, **b** 60 results for Obesity, **c** 391 results for Schizophrenia, and **d** 313 results for Autism. Detailed results are attached in Additional file 1

**Fig. 4:**
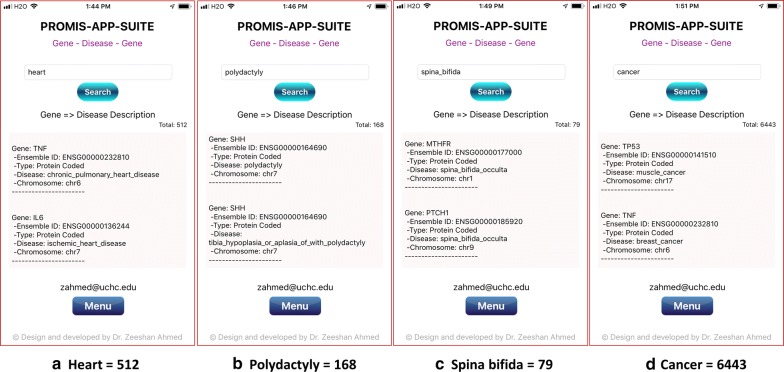
PAS-Gen screenshot examples of gene results (top two shown) from searches of the most common diseases: **a** 512 Heart and related diseases, **b** 168 results for Polydactyly, **c** 79 results for Spina Bifida, and **d** 6443 results for Cancer. Detailed results are attached in Additional file 1

**Fig. 5:**
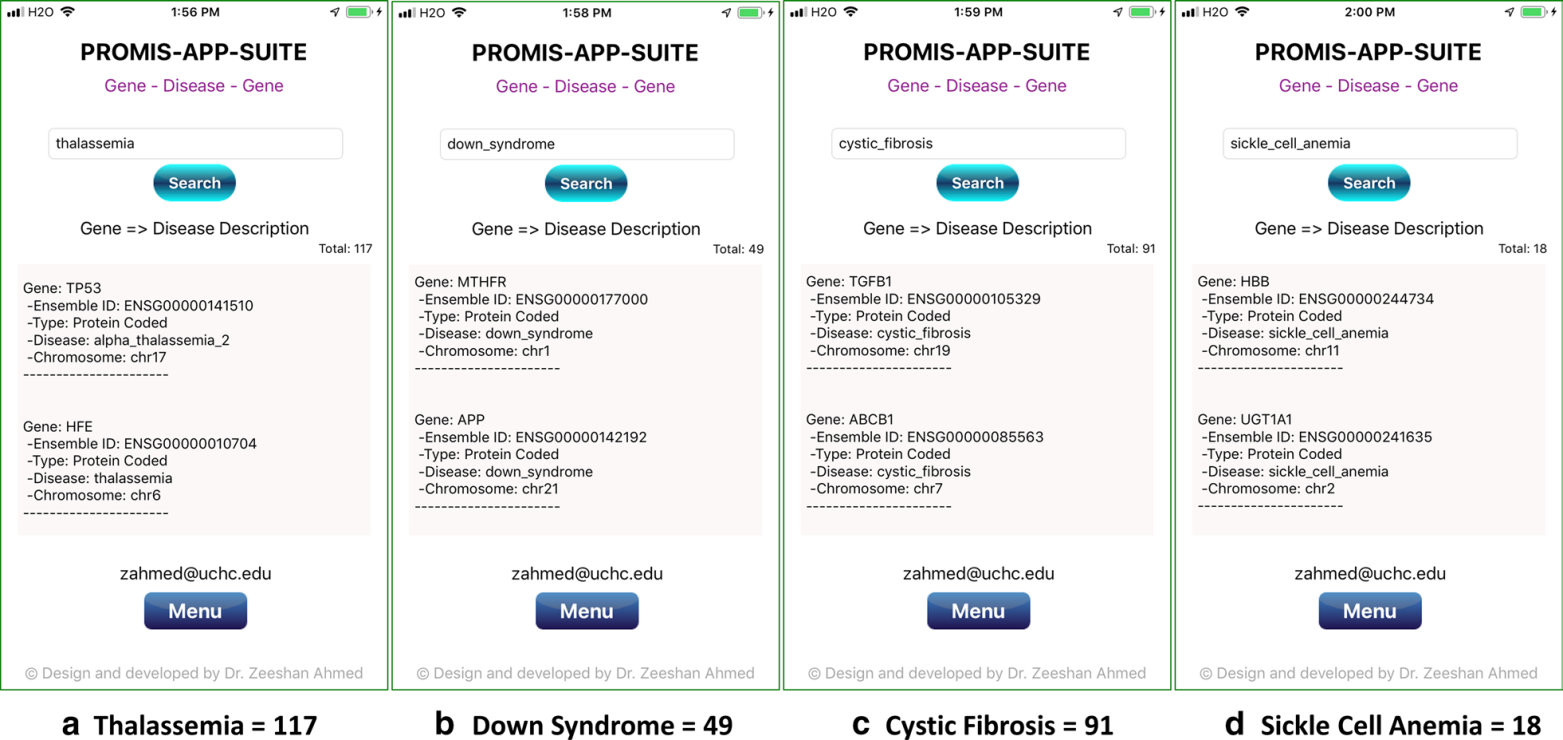
PAS-Gen screenshots examples of gene results (top two shown) for searches of common genetic diseases: **a** 117 results for Thalassemia, **b** 49 results for Down syndrome, **c** 91 results for Cystic Fibrosis, and **d** 18 results for Sickle Cell Anemia. Detailed results are attached in Additional file 1

**Fig. 6:**
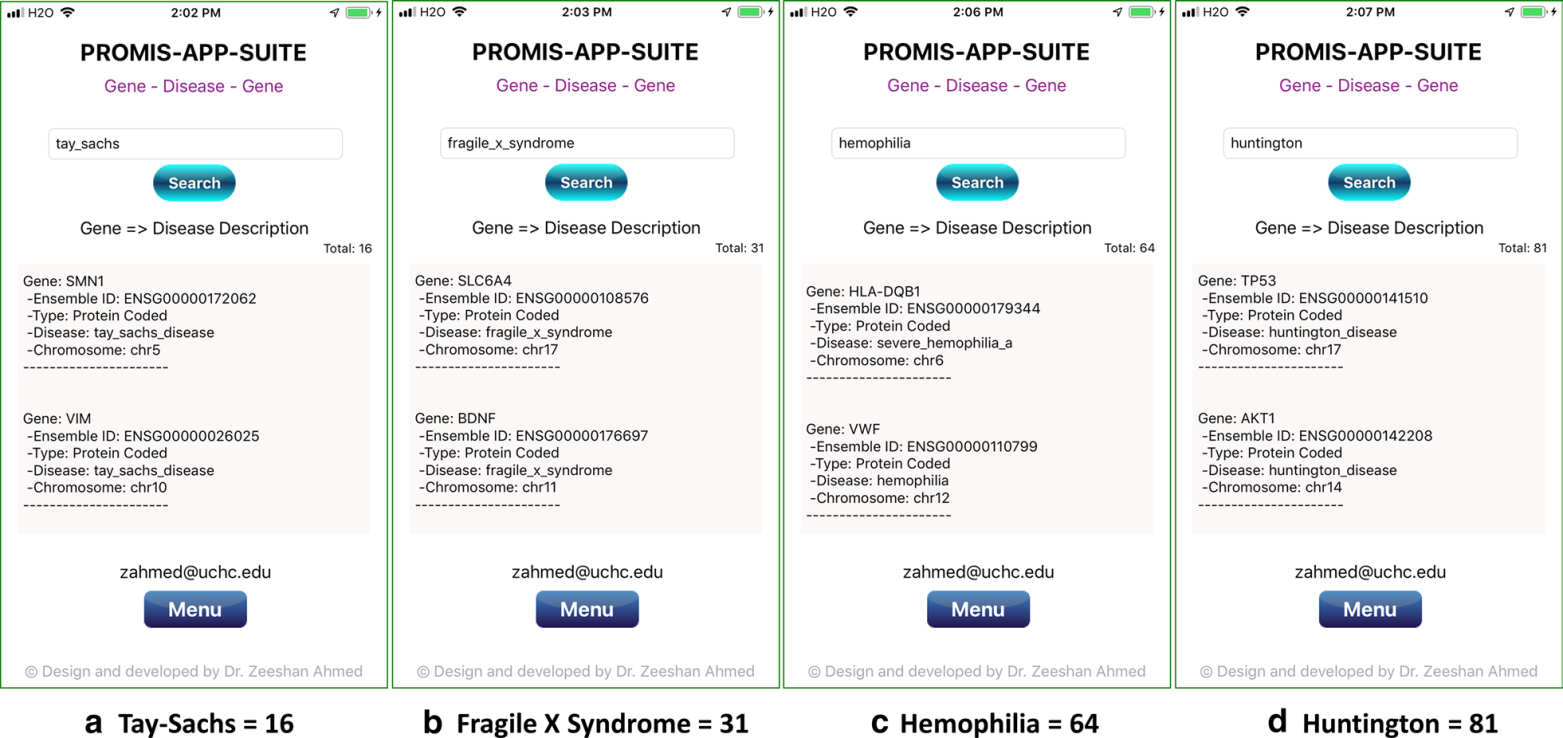
PAS-Gen screenshots examples of gene results (top two shown) for searches of common genetic diseases: **a** 16 results for Tay-Sachs disease, **b** 31 results for Fragile X Syndrome, **c** 64 results for Hemophilia, and **d** 81 results for Huntington. Detailed results are attached in Additional file 1

PAS-Gen is an easy-to-use application designed to simplify navigation across the landscape of gene annotation resources by an efficient mobile record search engine, which is based on standardized genes and related diseases to help explore multi-purpose clinical and genomics concepts in meaningful ways (Fig. 1). The PAS-Gen database includes a total of 59,293 genes, where 19,989 are protein-coding and 39,304 are non-protein-coding (processed transcript, lincRNA, antisense, IG C gene, bidirectional promoter lncRNA, polymorphic pseudogene, transcribed unitary pseudogene, transcribed unprocessed pseudogene, transcribed processed pseudogene, sense overlapping, scRNA, noncoding, unprocessed pseudogene, IG V gene, unitary pseudogene, vaultRNA, TR C gene, sense intronic, snRNA, processed pseudogene, TEC, TR V pseudogene, TR V gene, and macro lncRNA) (Table 1). The PAS-Gen database is composed of 98,064 gene-disease combinations reported from 809 distinct sources (combinations of sources for individual gene-disease relationship) and based on 26 types of genes, located at 23 pairs of genomic chromosomes and mitochondrial DNA, and 13,216 genes (including aliases), 10,598 genes with distinct Ensembl identifiers, 12,257 distinct diseases, 32,089 combinations with actionable genes, and 8063 cancer-causing genes (Table 2). Here, we present results to help users better understand the data search capabilities of PAS-Gen (Figs. 3, 4, 5, 6), detailed results are included in Additional file 2.

**Table 1:** PAS-Gen database description: type and sub-types of genes

#	Gene types	Gene sub-types
1	Protein coding	Coding
2	processed_transcript	non_coding
4	lincRNA	non_coding
5	Antisense	non_coding
6	IG_C_gene	non_coding
7	bidirectional_promoter_lncRNA	non_coding
8	polymorphic_pseudogene	non_coding
9	transcribed_unitary_pseudogene	non_coding
10	transcribed_unprocessed_pseudogene	non_coding
11	transcribed_processed_pseudogene	non_coding
12	sense_overlapping	non_coding
13	scRNA	non_coding
14	non_coding	non_coding
15	unprocessed_pseudogene	non_coding
16	IG_V_gene	non_coding
17	unitary_pseudogene	non_coding
18	vaultRNA	non_coding
19	TR_C_gene	non_coding
20	sense_intronic	non_coding
21	snRNA	non_coding
22	processed_pseudogene	non_coding
23	TEC	non_coding
24	TR_V_pseudogene	non_coding
25	TR_V_gene	non_coding
26	macro_lncRNA	non_coding

PAS-Gen database includes protein coding and 25 non-coding gene types (processed transcript, lincRNA, antisense, IG C gene, bidirectional promoter lncRNA, polymorphic pseudogene, transcribed unitary pseudogene, transcribed unprocessed pseudogene, transcribed processed pseudogene, sense overlapping, scRNA, non coding, unprocessed pseudogene, IG V gene, unitary pseudogene, vaultRNA, TR C gene, sense intronic, snRNA, processed pseudogene, TEC, TR V pseudogene, TR V gene, macro lncRNA)

**Table 2:** PAS-Gen database description and statistics

Categories	Count
Genes-disease combinations	98,064
Gene types	26
Chromosomes	24
Genes (including aliases)	13,216
Genes (Ensembl IDs)	10,598
Unique diseases	12,257
Genes-disease combinations based on actionable genes	32,089
Distinguished genes-disease source combinations	809
Cancer leading genes	8063

PAS-Gen database includes genes-disease combinations, gene types, chromosomes, genes (including aliases), genes (Ensembl IDs), diseases, actionable, source combinations, and cancer leading genes

A combination of various genetic and environmental factors leads to the most common diseases [39], e.g., Diabetes [40], Obesity [41], Schizophrenia [42, 43], Autism [44], Heart disease [45, 46], Polydactyly [47, 48], Spina Bifida [49], and Cancer [50]. The most common genetic diseases are Thalassemia [51], Down Syndrome [52], Cystic Fibrosis [53], Sickle Cell Anemia [54], Tay-Sachs disease [55], Fragile X Syndrome [56], Hemophilia [57], and Huntington [58]. Examples of gene search results for some of the most common diseases are shown in Figs. 3, 4 and the most common genetic diseases are shown in Figs. 5, 6. We present search results for gene-disease associations for the most common diseases, which includes 931 results for Diabetes, 60 results for Obesity, 391 results for Schizophrenia, 313 results for Autism, 512 Heart and related diseases, 168 results for Polydactyly, 79 results for Spina Bifida, and 6443 results for Cancer (Figs. 3, 4). Search results presenting gene-disease associations for most common genetic diseases include, 117 results for Thalassemia, 49 results for Down Syndrome, 91 results for Cystic Fibrosis, 18 results for Sickle Cell Anemia, 16 results for Tay-Sachs disease (Tay-Sachs is generally hyphenated, to search using PAS-Gen, its recommended to use underscore instead), 31 results for Fragile X Syndrome, 64 results for Hemophilia, and 81 results for Huntington (Figs. 5 and 6).

## Discussion

We are entering the era of personalized medicine in which an individual’s genetic makeup will eventually determine how a doctor can tailor his or her therapy. Therefore, it is critical to understand the genetic basis of common diseases (e.g., which genes and genetic variants contribute to disease phenotypes). Human diseases are at the heart of extensive research encompassing genomics, bioinformatics, systems biology, and systems medicine. To gain new insight into disease taxonomy, etiology, and pathogenesis, it’s important to understand how diseases are related to each other [29]. In the past, various efforts have been made in deciphering diseases to facilitate predictive diagnosis and thereby guide treatment factors [39], which includes drawing disease relationships using clinical manifestations [59–62], healthcare records [63–66], images and data generated using wearable technology and artificial intelligence [67–70], and information encapsulated within related genes [71, 72], proteins [73], signaling [74] and metabolic pathways [75], microRNA [76], chemo-centric views [77], phenotypic characteristics, and microbes [78]. Multiomics approaches (genome, transcriptome, proteome, metabolome, microbiome, and epigenome) are becoming increasingly common with the advancement of high-throughput technologies. A key challenge in this realm is NGS interpretation. Scientists are faced with the daunting challenge of identifying candidate genes that are relevant to their biological system of interest. Most often, the researcher only has direct knowledge of a few, if any, candidate genes. The clinical interpretation of the significance of specific gene variants can be unique to a patient. Variability in interpretation for sequence variants is due, in part, to the lack of standard curated information to support clinical decision-making.

The underlying assumption here is that creating a database with smart distillation and abundant distribution of genes and SNPs linked to the classified diseases and drugs through their description and IDs (e.g., ICD and NDC) can support both clinical and research environments [6]. Currently, investigation of multiple databases is required to assess the potential significance of even one sequence variant, and that is a cumbersome, time-consuming, and an increasingly unfeasible process with regard to identification and reports of variants in actionable genes because of the absence of a standard centralized platform for connecting genes to their disease phenotype [79]. Such a database must not be redundant and should only include human reference genome and disease-based information collected from valid sources available worldwide. It’s very important to facilitate interested users with efficient, user friendly, easy navigation, and free portable access to the database using platforms that have proven to be efficient tools in several areas including healthcare. In this manuscript, we present design and development of an iOS application to explore genes and diseases to support medical research that will support implementation of precision medicine.

The greatest strength of our approach is unearthing the biological roots of complex and rare diseases by facilitating mobile search mechanism for known and authentic genes that have been associated with their respective diseases. PAS-Gen aims to benefit every type of user (e.g., researchers, medical practitioners, life science students, and even patients) with easy one-touch browsing and saving time scanning through genes and developing gene-disease lists for a research study [6]. To harness the power of reported genes, our presented solution can contribute as a state-of-the-art, leading mobile application. In the future, we are looking to extend the scope of this project by curating and adding more genes, classified diseases and their relationships in PAS-Gen database, implementing data science and visualization features for analytics, and implementing actionable genes-based data classification e.g., The American College of Medical Genetics and Genomics (ACMG) [80] and MSK-IMPACT [81] approved actionable genes. We are extending the scope of our project by adding germline and somatic mutations, especially maintained by the Genome-Wide Association Studies (GWAS) [82] and Catalog of Somatic Mutations in Cancer (COSMIC) [83, 84]. We aim for the integration and annotation of our genomics (genes and variants) and clinical (diseases and drugs and their code sets) databases to assist clinicians to directly interpret a patient’s genomic profile and collaborate with scientists to translate variant data into therapy. Furthermore, we are interested in advancing the graphical user interface of PAS-Gen with the implementation of machine learning techniques to facilitate users in intelligently searching data of their interest based on their personal preferences and search history.

## Conclusions

Gene-disease data are highly significant at every level of biological research and healthcare, but inconsistencies and inabilities in terms of gene annotation and specificity of disease classification terminologies add to the complexity and lack of an efficient integrative searchable system make it difficult to comprehend the underlying implications. We offer PAS-Gen to the biomedical research community with a social pledge to educate individuals by providing them with an interactive app to query, easily explore, and access information on gene annotation and classified disease phenotypes with greater visibility and easy browsing. The gene-disease querying ability offered by PAS-Gen provides the user with an important knowledge discovery tool, just a click away from any location. PAS-Gen is an exclusively academic application founded on genomics, clinical, scientific, and modern technology to support healthcare by enabling scientific data retrieval using efficient mobile-based tools.

## Supplementary information

**Additional file 1.** Additional data reported in Figs. 3, 4, 5, 6.

**Additional file 2.** PAS-Gen: Guide to iOS app with gene-disease classifications.

## Data Availability

The datasets used and analyzed during the current study are available from the corresponding author on reasonable request, and can also be accessed using PAS-Gen app https://apps.apple.com/us/app/pas-gen/id1447766164?ls=1.

## Abbreviations

ACMG: American College of Medical Genetics and Genomics
BRCA: BReast CAncer gene
DNA: deoxyribonucleic acid
ICD: International Classification of Diseases
NGS: next generation sequencing
PAS: PROMIS-APP-SUITE
PLA: product line architecture
SNPs: single nucleotide polymorphisms
TTN: titin
WHO: World Health Organization

## References

[1] He KY, Ge D, He MM (2017). Big data analytics for genomic medicine. Int J Mol Sci.

[2] Escalona M, Rocha S, Posada D (2016). A comparison of tools for the simulation of genomic next-generation sequencing data. Nat Rev Genet.

[3] Miller HI, Konkel DA, Leder P (1978). An intervening sequence of the mouse beta-globin major gene shares extensive homology only with beta-globin genes. Nature.

[4] Friedmann T (1992). A brief history of gene therapy. Nat Genet.

[5] Maglott D (2004). Entrez Gene: gene-centered information at NCBI. Nucleic Acids Res.

[6] Zeeshan S, Xiong R, Liang BT, Ahmed Z (2019). 100 years of evolving gene-disease complexities and scientific debutants. Briefings Bioinform.

[7] Laird CD (1971). Chromatid structure: relationship between DNA content and nucleotide sequence diversity. Chromosoma.

[8] Alberts B, Johnson A, Lewis J (2003). Molecular biology of the cell. Ann Bot.

[9] Flavell RA, Glover DM, Jeffreys AJ (1978). Discontinuous genes. Trends Biochem Sci.

[10] LeWinter MM, Granzier HL (2013). Titin is a major human disease gene. Circulation.

[11] Lander ES (2011). Initial impact of the sequencing of the human genome. Nature.

[12] Brunham LR, Hayden MR (2013). Hunting human disease genes: lessons from the past, challenges for the future. Hum Genet.

[13] Abbott A (2010). Project set to map marks on genome. Nature.

[14] Frazer KA (2012). Decoding the human genome. Genome Res.

[15] Falk MJ, Sondheimer N (2010). Mitochondrial genetic diseases. Curr Opin Pediatr.

[16] Lobo I, Zhaurova K (2008). Birth defects: causes and statistics. Nat Educ..

[17] Chial H (2008). Mendelian genetics: patterns of inheritance and single-gene disorders. Nat Educ..

[18] Kibbe WA, Arze C, Felix V (2014). Disease Ontology 2015 update: an expanded and updated database of human diseases for linking biomedical knowledge through disease data. Nucleic Acids Res.

[19] Zhang G, Shi J, Zhu S (2017). DiseaseEnhancer: a resource of human disease-associated enhancer catalog. Nucleic Acids Res.

[20] Pletscher-Frankild S, Pallejà A, Tsafou K (2015). DISEASES: Text mining and data integration of disease-gene associations. Methods.

[21] Piñero J, Pallejà A, Tsafou K (2017). DisGeNET: A comprehensive platform integrating information on human disease-associated genes and variants. Nucleic Acids Res.

[22] Babbi G, Martelli PL, Profiti G (2017). eDGAR: A database of disease-gene associations with annotated relationships among genes. BMC Genomics..

[23] Safran M, Dalah I, Alexander J (2010). GeneCards Version 3: the human gene integrator. Database..

[24] Rubinstein WS, Maglott DR, Lee JM (2012). The NIH genetic testing registry: a new, centralized database of genetic tests to enable access to comprehensive information and improve transparency. Nucleic Acids Res.

[25] Rappaport N, Twik M, Nativ N (2014). MalaCards: a comprehensive automatically-mined database of human diseases. Curr Protoc Bioinform..

[26] Amberger JS, Bocchini CA, Schiettecatte F, Scott AF, Hamosh A (2014). OMIM.org: Online Mendelian Inheritance in Man (OMIM^®^), an online catalog of human genes and genetic disorders. Nucleic Acids Res.

[27] Jiang Q, Wang Y, Hao Y (2008). miR2Disease: a manually curated database for microRNA deregulation in human disease. Nucleic Acids Res.

[28] Stenson PD, Mort M, Ball EV (2017). The Human Gene Mutation Database: towards a comprehensive repository of inherited mutation data for medical research, genetic diagnosis and next-generation sequencing studies. Hum Genet.

[29] Yang J, Wu SJ, Yang SY (2016). DNetDB: The human disease network database based on dysfunctional regulation mechanism. BMC Syst Biol.

[30] Landrum MJ, Lee JM, Benson M (2015). ClinVar: public archive of interpretations of clinically relevant variants. Nucleic Acids Res.

[31] Qiu F, Xu Y, Li K (2012). CNVD: Text mining-based copy number variation in disease database. Hum Mutat.

[32] Cunningham F, Achuthan P, Akanni W (2018). Ensembl 2019. Nucleic Acids Res.

[33] Frankish A, Diekhans M, Ferreira AM (2018). GENCODE reference annotation for the human and mouse genomes. Nucleic Acids Res.

[34] Ahmed Z (2009). Proposing semantic oriented agent and knowledge base product data management. Inf Manag Comput Secur..

[35] Ahmed Z (2010). Towards performance measurement and metrics based analysis of PLA applications. Int J Softw Eng Appl..

[36] Ahmed Z (2011). Designing flexible GUI to increase the acceptance rate of product data management systems in industry. Int J Comput Sci Emerg Technol..

[37] Ahmed Z, Zeeshan S, Dandekar T (2014). Developing sustainable software solutions for bioinformatics by the “Butterfly” paradigm. F1000Research..

[38] Ahmed Z, Zeeshan S (2014). Cultivating software solutions development in the scientific academia. Recent Patents Comput Sci..

[39] Karczewski KJ, Snyder MP (2018). Integrative omics for health and disease. Nat Rev Genet.

[40] Flannick J, Florez JC (2016). Type 2 diabetes: genetic data sharing to advance complex disease research. Nat Rev Genet.

[41] Locke AE, Kahali B, Berndt SI (2015). Genetic studies of body mass index yield new insights for obesity biology. Nature.

[42] Schizophrenia Working Group of the Psychiatric Genomics Consortium (2014). Biological insights from 108 schizophrenia-associated genetic loci. Nature.

[43] Fromer M, Roussos P, Sieberts SK (2016). Gene expression elucidates functional impact of polygenic risk for schizophrenia. Nat Neurosci.

[44] Grove J, Ripke S, Damm T (2017). Common risk variants identified in autism spectrum disorder. bioRxiv..

[45] Benjamin EJ, Blaha MJ, Chiuve SE (2017). Heart disease and stroke statistics—2017 update: a report from the American heart association. Circulation.

[46] Stewart J, Manmathan G, Wilkinson P (2017). Primary prevention of cardiovascular disease: a review of contemporary guidance and literature. JRSM Cardiovasc Dis..

[47] Umair M, Ahmad F, Bilal M, Ahmad W, Alfadhel M (2018). Clinical genetics of polydactyly: an updated review. Front Genet..

[48] Ahmed H, Akbari H, Emami A, Akbari MR (2017). Genetic overview of syndactyly and polydactyly. Plast Reconstr Surg Glob Open..

[49] Copp AJ, Adzick NS, Chitty LS (2015). Spina bifida. Nat Rev Dis Primers..

[50] Blackadar CB (2016). Historical review of the causes of cancer. World J Clin Oncol..

[51] Marengo-Rowe AJ (2007). The thalassemias and related disorders. Bayl Univ Med Cent Proc.

[52] Kazemi M, Salehi M, Kheirollahi M (2016). Down syndrome: current status, challenges and future perspectives. Int J Mol Cell Med..

[53] Davies JC, Alton EW, Bush A (2007). Cystic fibrosis. BMJ.

[54] Ilesanmi OO (2010). Pathological basis of symptoms and crises in sickle cell disorder: implications for counseling and psychotherapy. Hematol Rep..

[55] Solovyeva VV, Shaimardanova AA, Chulpanova DS (2018). New approaches to Tay-Sachs disease therapy. Front Physiol..

[56] Saldarriaga W, Tassone F, González-Teshima LY (2014). Fragile X syndrome. Colomb Med.

[57] Coppola A, Di Capua M, Di Minno MN (2010). Treatment of hemophilia: a review of current advances and ongoing issues. J Blood Med..

[58] Roos RA (2010). Huntington’s disease: a clinical review. Orphanet J Rare Dis..

[59] van Driel MA, Bruggeman J, Vriend G (2006). A text-mining analysis of the human phenome. Eur J Hum Genet.

[60] Lage K, Karlberg EO, Storling ZM (2007). A human phenome-interactome network of protein complexes implicated in genetic disorders. Nat Biotechnol.

[61] Kohler S, Doelken SC, Mungall CJ (2014). The Human Phenotype Ontology project: linking molecular biology and disease through phenotype data. Nucleic Acids Res.

[62] Zhou X, Menche J, Barabasi AL, Sharma A (2014). Human symptoms-disease network. Nat Commun..

[63] Blair DR, Lyttle CS, Mortensen JM (2013). A nondegenerate code of deleterious variants in Mendelian loci contributes to complex disease risk. Cell.

[64] Jensen AB, Moseley PL, Oprea TI (2014). Temporal disease trajectories condensed from population-wide registry data covering 6.2 million patients. Nat Commun..

[65] Davis DA, Chawla NV (2011). Exploring and exploiting disease interactions from multi-relational gene and phenotype networks. PLoS ONE.

[66] Hidalgo CA, Blumm N, Barabasi AL, Christakis NA (2009). A dynamic network approach for the study of human phenotypes. PLoS Comput Biol.

[67] Jiang F, Jiang Y, Zhi H (2017). Artificial intelligence in healthcare: past, present and future. Stroke Vasc Neurol..

[68] Wahl B, Cossy-Gantner A, Germann S, Schwalbe NR (2018). Artificial intelligence (AI) and global health: how can AI contribute to health in resource-poor settings?. BMJ Glob Health..

[69] Guo J, Li B (2018). The application of medical artificial intelligence technology in rural areas of developing countries. Health Equity..

[70] Jones LD, Golan D, Hanna SA, Ramachandran M (2018). Artificial intelligence, machine learning and the evolution of healthcare: a bright future or cause for concern?. Bone Jt Res..

[71] Goh KI, Cusick ME, Valle D (2007). The human disease network. Proc Natl Acad Sci USA..

[72] Liu YI, Wise PH, Butte AJ (2009). The “etiome”: identification and clustering of human disease etiological factors. BMC Bioinform.

[73] Hamaneh MB, Yu YK (2015). DeCoaD: determining correlations among diseases using protein interaction networks. BMC Res Notes..

[74] Li Y, Agarwal P (2009). A pathway-based view of human diseases and disease relationships. PLoS ONE.

[75] Lee DS, Park J, Kay KA, Christakis NA, Oltvai ZN, Barabasi AL (2008). The implications of human metabolic network topology for disease comorbidity. Proc Natl Acad Sci USA.

[76] Lu M, Zhang Q, Deng M, Miao J, Guo Y, Gao W, Cui Q (2008). An analysis of human microRNA and disease associations. PLoS ONE.

[77] Duran-Frigola M, Rossell D, Aloy P (2014). A chemo-centric view of human health and disease. Nat Commun..

[78] Ma W, Zhang L, Zeng P, Huang C, Li J, Geng B, Yang J, Kong W, Zhou X, Cui Q (2016). An analysis of human microbe-disease associations. Brief Bioinform..

[79] Biesecker LG, Nussbaum RL, Rehm HL (2018). Distinguishing variant pathogenicity from genetic diagnosis: how to know whether a variant causes a condition. JAMA.

[80] Richards S, Aziz N, Bale S (2015). Standards and guidelines for the interpretation of sequence variants: a joint consensus recommendation of the American College of Medical Genetics and Genomics and the Association for Molecular Pathology. Genet Med..

[81] Cheng DT, Mitchell TN, Zehir A (2015). Memorial Sloan Kettering-Integrated Mutation Profiling of Actionable Cancer Targets (MSK-IMPACT): a hybridization capture-based next-generation sequencing clinical assay for solid tumor molecular oncology. J Mol Diagn..

[82] Ku CS (2010). The discovery of human genetic variations and their use as disease markers: past, present and future. J Hum Genet.

[83] Bamford S (2004). The COSMIC (Catalogue of Somatic Mutations in Cancer) database and website. Br J Cancer.

[84] Tate JG (2018). COSMIC: the Catalogue Of Somatic Mutations In Cancer. Nucleic Acids Res.

