# Supplementary material for: Debutant iOS app and gene-disease complexities in clinical genomics and precision medicine
Source: Clin Transl Med. 2019 Oct 4;8:26. doi: 10.1186/s40169-019-0243-8 (PMC6778157; doi:10.1186/s40169-019-0243-8)
Supplement: Supplementary file 2 — Additional file 2. PAS-Gen: Guide to iOS app with gene-disease classifications. [file 40169_2019_243_MOESM2_ESM.pdf]

*Additional file# 2: Debutant iOS app and gene-disease complexities in clinical genomics and precision medicine.*

# PAS-Gen: Guide to iOS app with gene-disease classifications

Zeeshan Ahmed[1, 2, *], Saman Zeeshan[3], Ruoyun Xiong[1, 3], Bruce T. Liang[4]

[1]Department of Genetics and Genome Sciences, School of Medicine, University of Connecticut Health Center (UConn Health), 263 Farmington Ave, Farmington, CT, 06032, USA.

[2]Institute for Systems Genomics, University of Connecticut, 263 Farmington Ave, Farmington, CT, 06032, USA.

[3]The Jackson Laboratory for Genomic Medicine, 10 Discovery Drive, Farmington, CT, 06032, USA.

[4]Pat and Jim Calhoun Cardiology Center, School of Medicine, UConn Health, 263 Farmington Ave, Farmington, CT, 06032, USA.

**[*]Corresponding author:**

Zeeshan Ahmed

Email: zahmed@uchc.edu

Lab URL: https://health.uconn.edu/ahmed-lab/

Mail: Department of Genetics and Genome Sciences, School of Medicine, University of Connecticut Health Center (UConn Health), 263 Farmington Ave, Farmington, CT, 06032, USA.

# PAS-Gen

PROMIS-APP-SUITE (PAS) - Gen is an iOS app to assist clinical and life science's researchers by providing larger exposure to the authentic genes and their association to classified diseases with greater visibility and easy one tap browsing, saving time in scanning through genes and developing gene-disease lists for a research study. PAS-Gen is an app developed on iPhone Operating System (iOS) platform (version 12.1). PAS-Gen's graphical user interface are developed using Swift multi-paradigm programming language and XCODE (version 10) integrated development environment for macOS. The database is modelled and hosted within MySQL database management system. It has dynamic, web based pages to facilitate data migration between app and database are developed PHP scripting language (Figure S1).

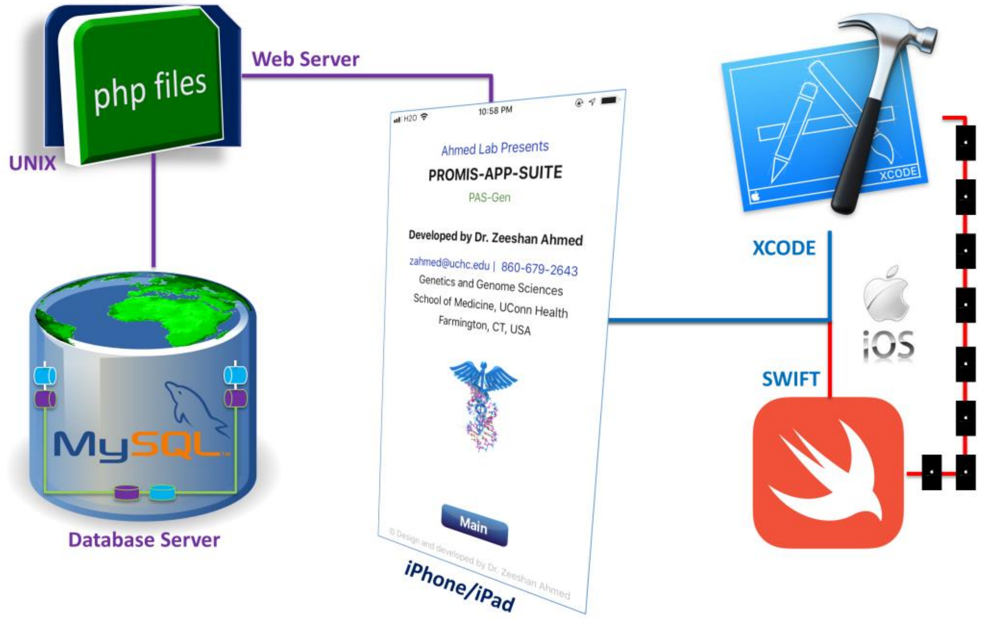


**Figure S1.** PAS-Gen components design, development, and data flow.

PAS-Gen is based on product line architecture (PLA), designed following the Butterfly model [1, 2, 3], with all major modules capable of performing individual key roles and can integrate with each other. One of the most difficult and complex tasks of implementing an Apple mobile app connecting via PHP programmed modules to an external web-based MySQL server for data exchange, is the integration of all modules developed using different programming languages and processed through different compilers/interpreters that sometimes cause non-syntax logical errors, which are hard to debug. Its normalized relational database includes over 59,000 genes and over 90,000 gene-disease combinations collected from various databases worldwide, including human reference genome from Ensembl [4] and GenCode [5].

PAS-Gen was mainly tested using XCODE-provided simulator and Apple iPhone 8, X and iPad mobile devices with the most recent iOS version (12.1). It is reviewed and approved by the Apple, and freely available to download at the App Store <https://itunes.apple.com/us/app/pas-gen/id1447766164?ls=1&mt=8>.

# PAS-Gen: Main

This is the first launched, main (Figure S2) user interface of the PAS-Gen app, leading to five other sub-interfaces for taking different inputs from the user, allowing user to perform different operations and sharing important information.

Main interface provides following six features:

1. At the launch of the app, “Main” automatically checks if user has the latest version of app. In case of an outdated, obsolete version of the app, it will automatically navigate user to the message interface (Figure S3), which will guide user to the available newer version.
2. Main allows user to navigate to the “About” interface (Figure S2) by pressing button “About”, which provides information about the app and author’s contacts.
3. PAS-Gen requires internet as a mandatory requirement, Main allows user to check if the iPhone device is successfully connected to the available internet service (Figure S3) by pressing button “CiC”.
4. Main allows user to navigate to user registration interface (Figure S4) by pressing “Register” button.
5. Main allows user to navigate to change password interface (Figure S4) by pressing “Reset/Forget Password” button.
6. Main allows user to enter user name and password, and press “Login” button. Having valid user credentials, it automatically navigates to the Menu interface (Figure S5).

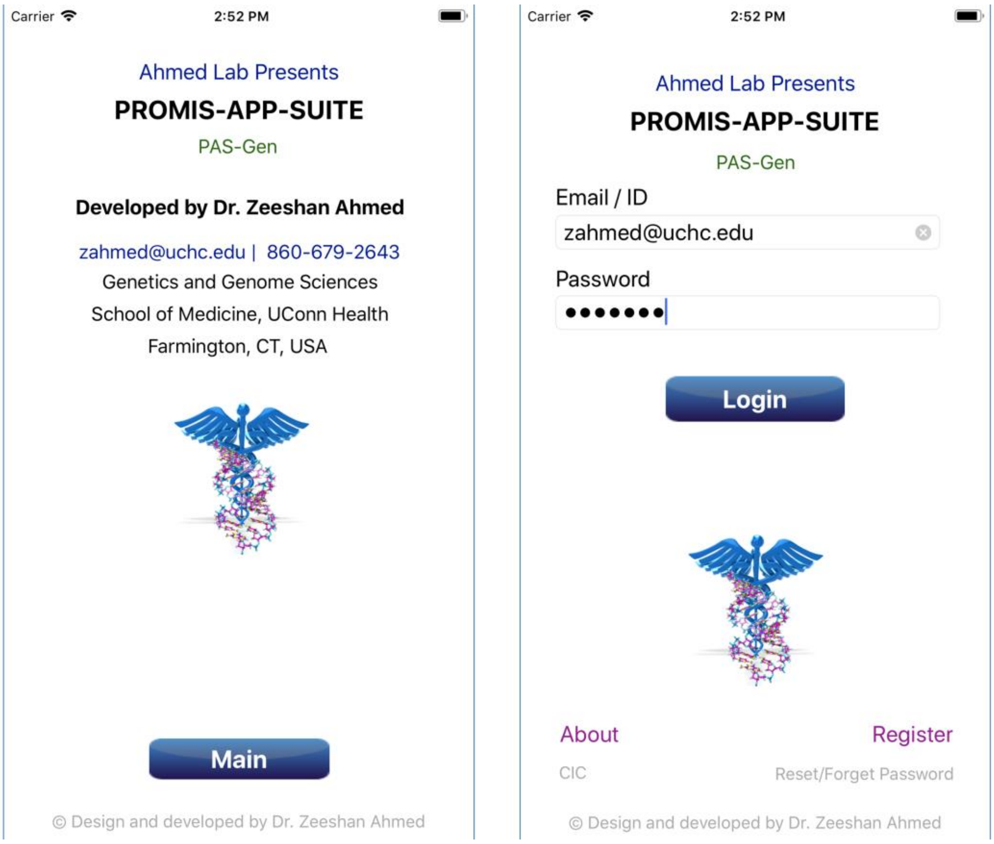


**Figure S2.** PAS-Gen About and Main interfaces.

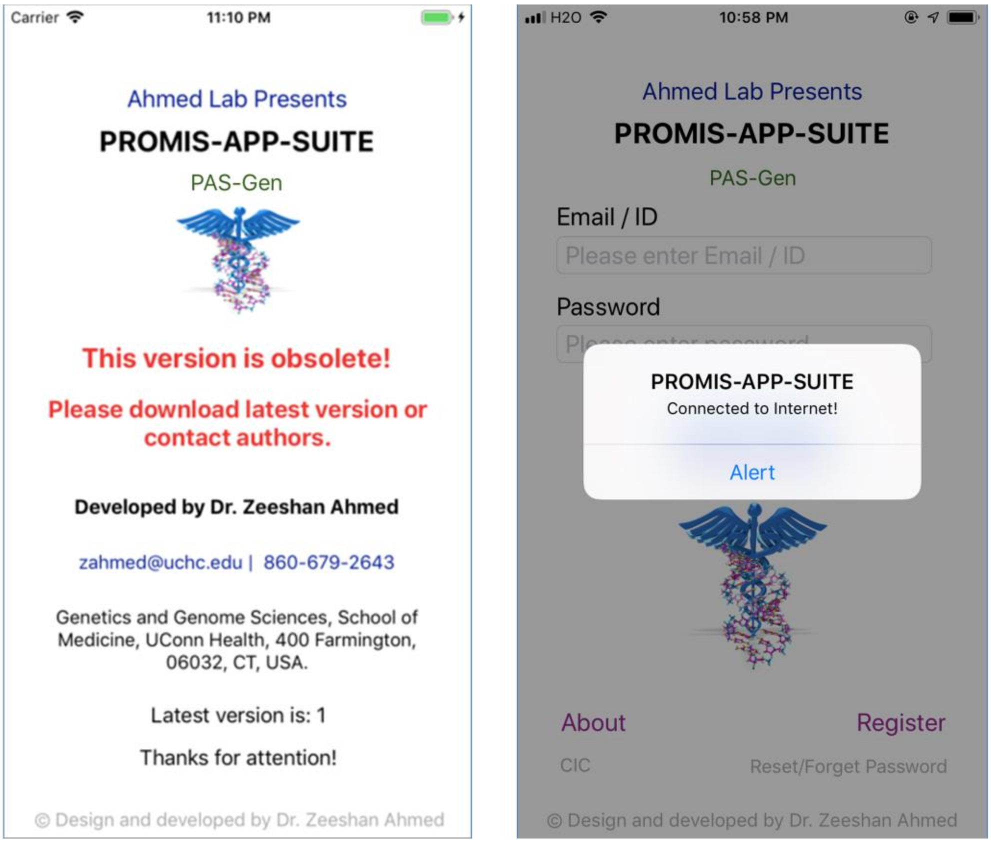


**Figure S3.** PAS-Gen obsolete version and Check Internet Connection (CIC) prompt.

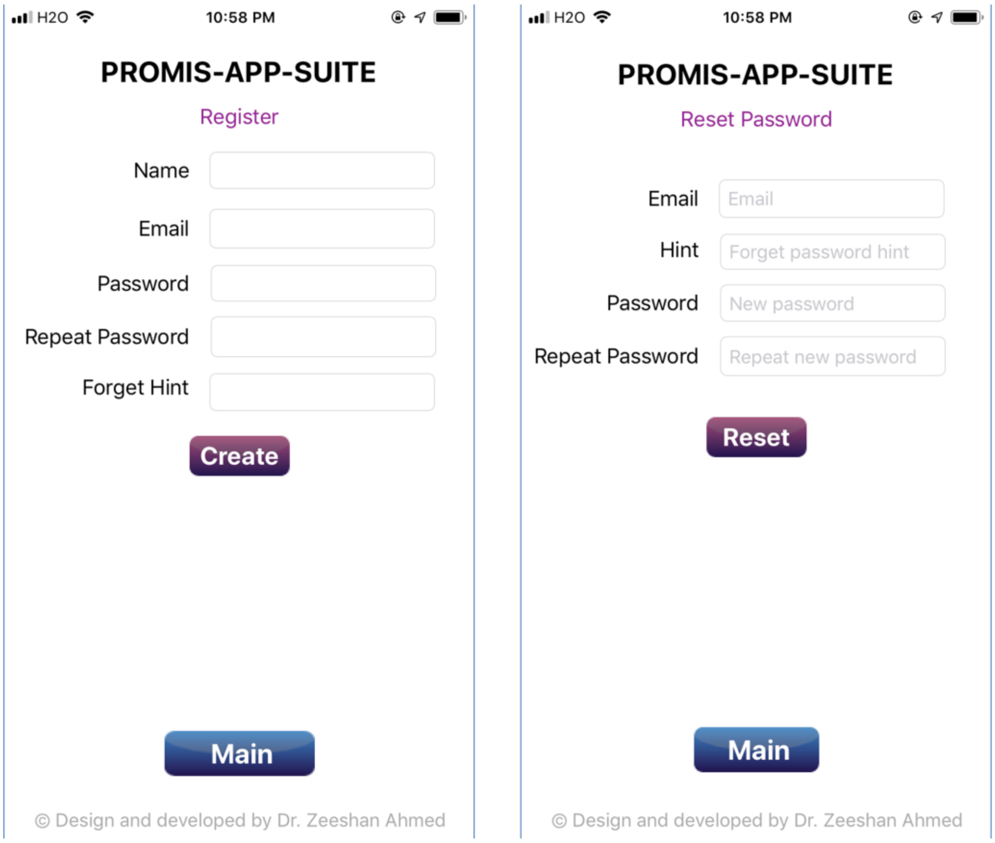


**Figure S4.** PAS-Gen Create new user and reset password

# PAS-Gen: Register & Password Reset

Register (Figure S4) interface allows user to create new login for using PAS-Gen. It requires user to enter seven following values:

1. Full name of the user.
2. Email of the user, which will also be the user's ID for PAS-Gen.
3. Password
4. Repeat password to make sure, user's entered is the user's expected password.
5. Forget hint, to use when password is forgotten and need to reset password.

After entering requested information, user is required to press "Create" button to register, and press "Main" button to navigate back to the Main interface.

Reset Password (Figure S4) interface allows user to reset the password. It requires user to enter following four data entries:

1. Email address, entered by the user to register.
2. Forget password hint, entered at the time user registered.
3. New password.
4. Repeat password to make sure, user's entered is the user's expected password.

After entering requested information, user is required to press "Reset" button to set new password, and press "Main" button to navigate back to the Main interface.

# PAS-Gen: Menu

Menu interface allows user to navigate to Genomics and Clinical Genomics interfaces (Figure S5). Genomic further navigates to Genes interface, whereas, Clinical Genomics further navigates to Gene-Disease interface (Figure S6). Moreover, Menu provides "Logout" button to sign out and navigate back to the Main interface, and Genomics and Clinical Genomics provides "Menu" button to navigate back to the Menu interface.

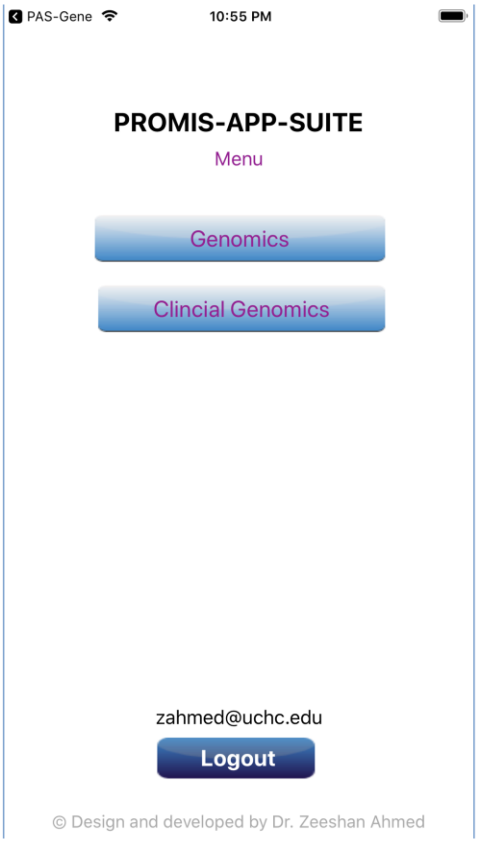


**Figure S5.** PAS-Gen Menu: Genomics and Clinical Genomics

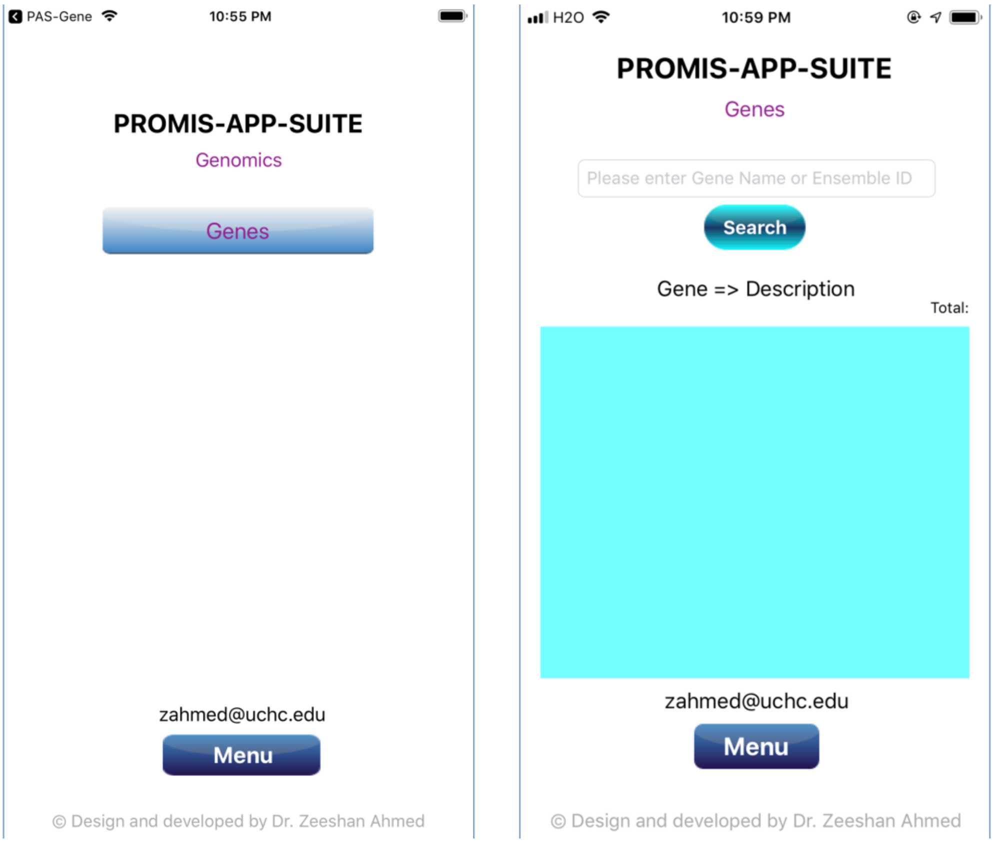


**Figure S6.** Navigating from PAS-Gen Menu to Gene to Disease interface

# PAS-Gen: Genes

Genes (Figure S7) provides white input text field and allows user to enter complete or partial gene name or ensemble id and search by pressing “Search” button. The results are presented in the green text box, and the total number of relevant records found are provided in the text field below. Moreover, Genes provides “Menu” button to navigate back to the Menu interface. The Genes’ interface enables users to search for Genes and related details, which includes: Gene Name, Ensemble ID, Type and Chromosome. The PAS-Gen graphical user interface enables users to search by complete or partial word matching.

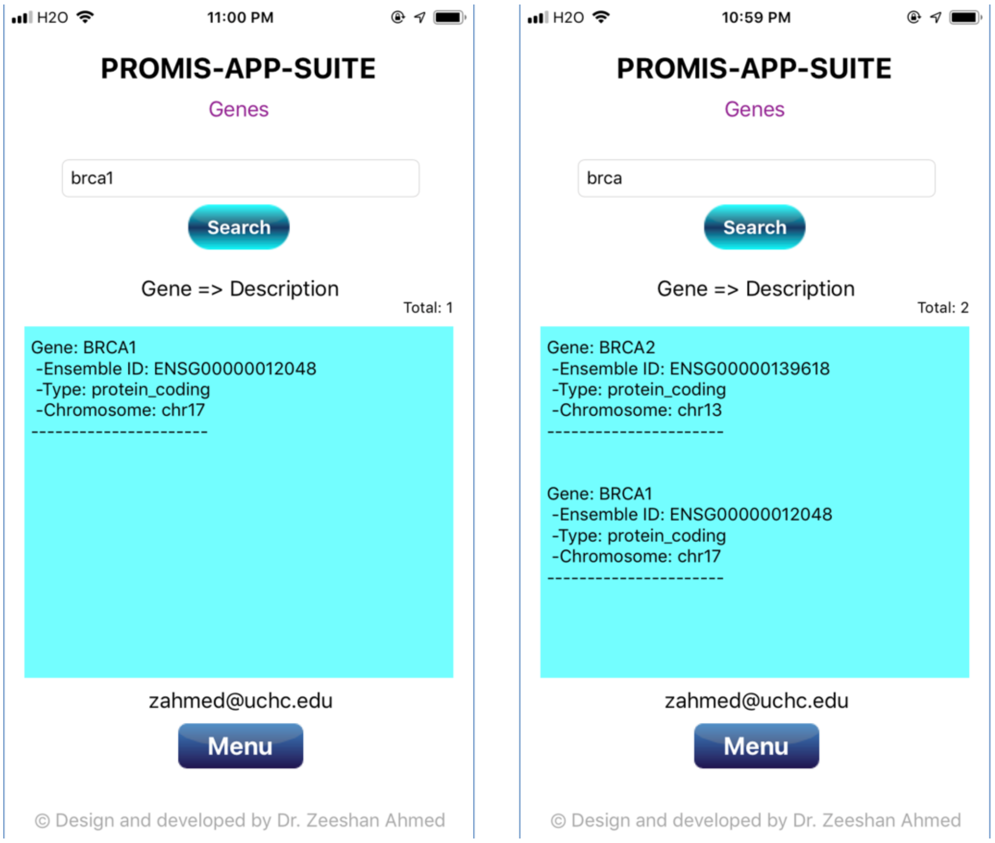


**Figure S7.** PAS-Gen Genes’ interface with examples searches, which include full gene name “BRCA1” and partial gene name “BRCA”.

# PAS-Gen: Gene to Disease

User needs to navigate from Menu to Clinical Genomics, and then to Gene to Disease interface (Figure S8). Gene to Disease provides a white input text field and allows user to enter complete or partial gene and disease name and search by pressing “Search” button.

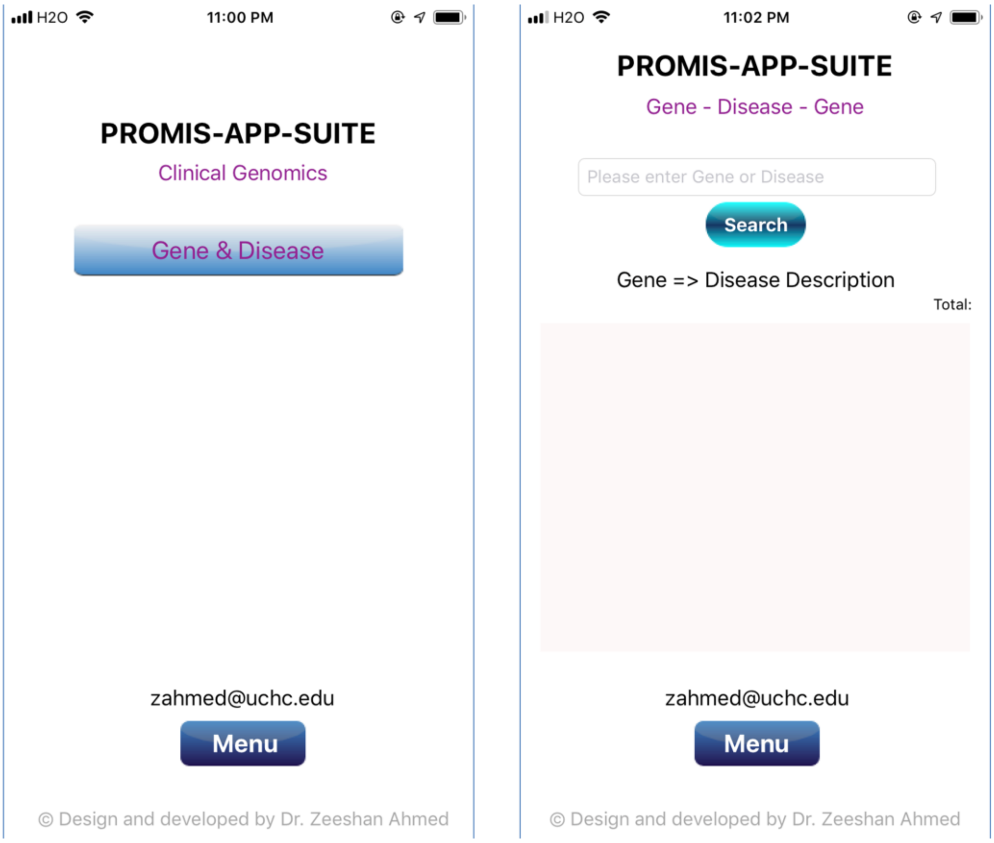


**Figure S8.** Navigating from PAS-Gen Menu to Gene to Disease interface

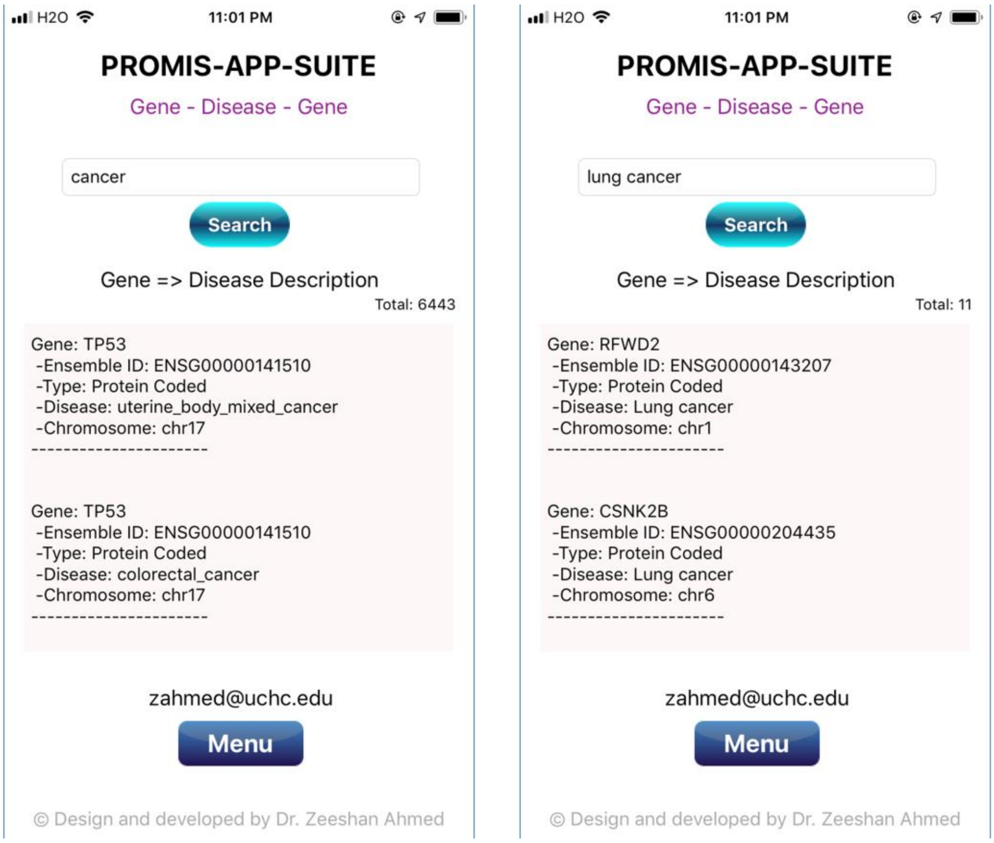


**Figure S9.** PAS-Gen Gene to Disease

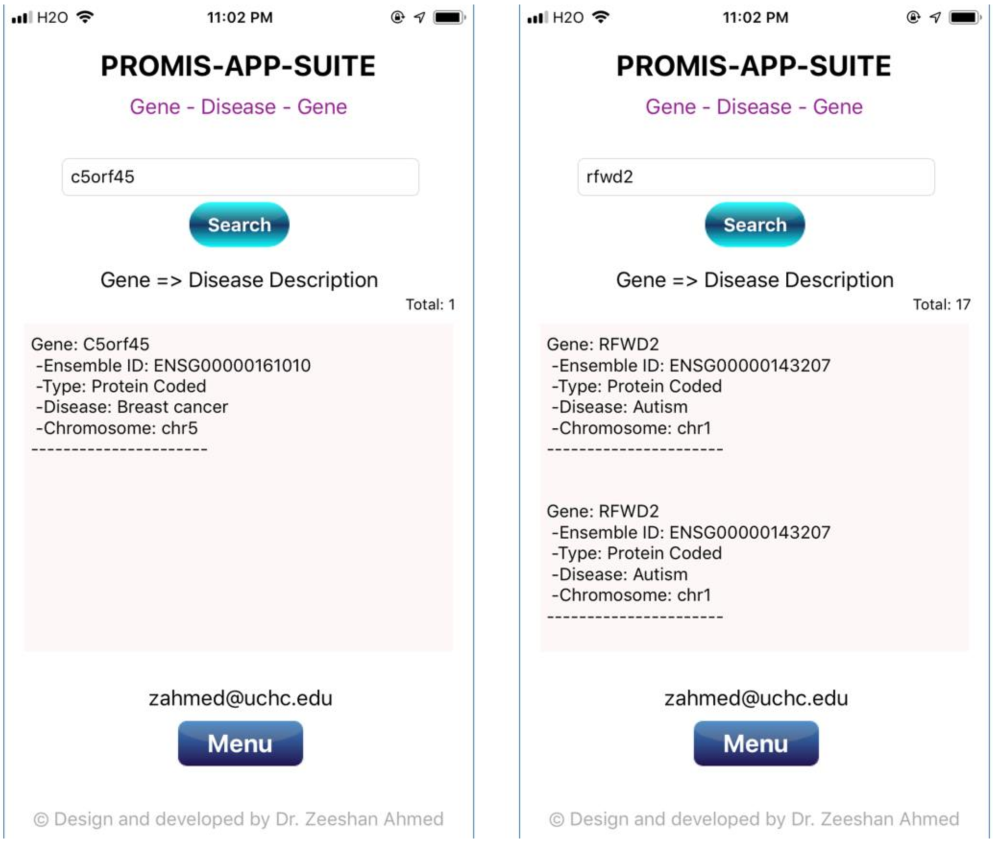


**Figure S10.** PAS-Gen Gene to Disease interface with examples searches, which include gene names “cforf45” and “rfwd2”.

The results are presented in red text box, and the total number of relevant records found are given in below text field. Moreover, Gene to Disease provides “Menu” button to navigate back to the Menu interface. The Genes to Disease’ interface enables users to search for Genes, Disease and related details, which includes: Gene Name, Ensemble ID, Type, Disease and Chromosome.

The PAS-Gen graphical user interface enables users to search by complete or partial word matching (Figure S9 and Figure S10). The gene-disease querying ability offered by PAS-Gen provides the user with an important knowledge discovery tool, just a click away from any location.

# PAS-Gen: Download

PAS-Gen is freely available to download at App Store (Figure S11), and is recommended for iPhone 8, X and iPad mobile devices with iOS version 12.1.

Project App Store are available (Figure S12) at: <https://itunes.apple.com/us/app/pas-gen/id1447766164?ls=1&mt=8>.

Project website <https://health.uconn.edu/ahmed-lab/projects/pas/pas-gen/>.

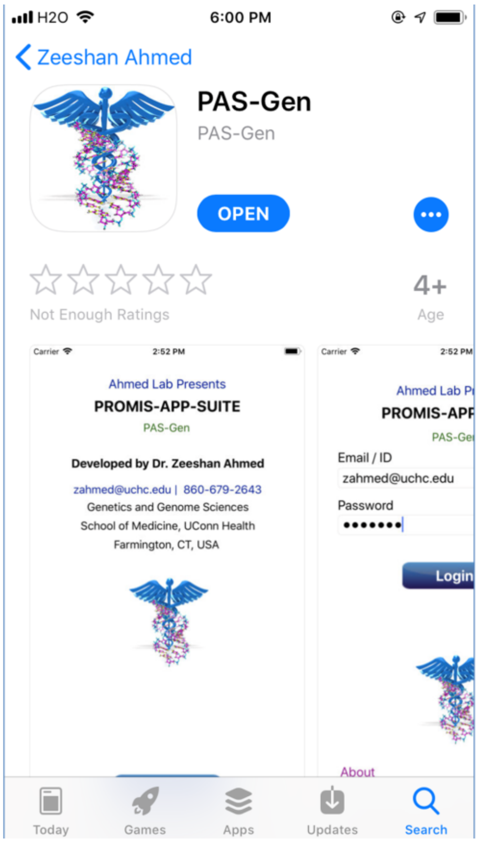


**Figure S11.** Downloading PAS-Gen from App Store (iPhone 8 interface).

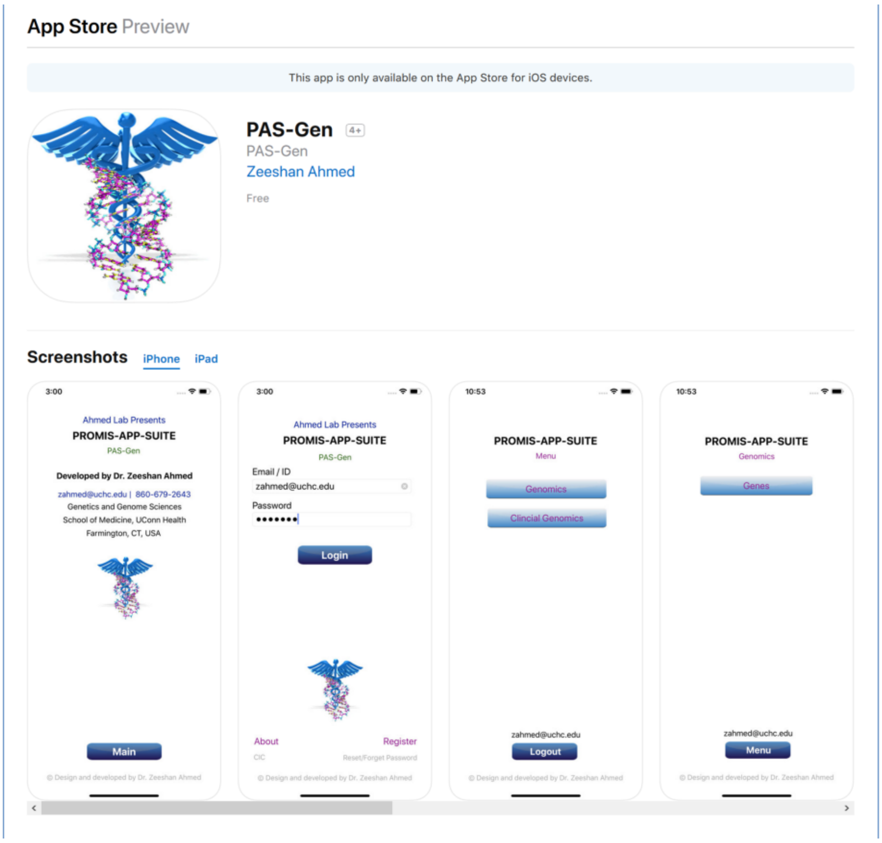


**Figure S12.** Downloading PAS-Gen from App Store (Web interface).

# References


1. Ahmed Z, Zeeshan S, Dandekar T. Developing sustainable software solutions for bioinformatics by the “Butterfly” paradigm. F1000Research. 2014;7:54-66.

2. Ahmed Z, Zeeshan S. Cultivating Software Solutions Development in the Scientific Academia. Recent Patents on Computer Science. 2014;7:54-66.

3. Ahmed Z. Designing Flexible GUI to Increase the Acceptance Rate of Product Data Management Systems in Industry. International Journal of Computer Science & Emerging Technologies. 2011;2:100-109.

4. Cunningham F, Achuthan P, Akanni W, et al. Ensembl 2019. Nucleic Acids Res. 2018;47(D1):D745-D751.

5. Frankish A, Diekhans M, Ferreira AM, et al. GENCODE reference annotation for the human and mouse genomes. Nucleic Acids Res. 2018;47(D1):D766-D773.
